# Endocrine therapy in advanced high-grade ovarian cancer: real-life data from a multicenter study and a review of the literature

**DOI:** 10.1093/oncolo/oyae093

**Published:** 2024-05-20

**Authors:** Marine Aubert, Laurent Mathiot, Hélène Vegas, Lobna Ouldamer, Claude Linassier, Paule Augereau, François Bocquet, Jean-Sébastien Frenel, Mathilde Cancel

**Affiliations:** Department of Medical Oncology, CHU Bretonneau Tours, Tours, France; Department of Medical Oncology, Institut de Cancérologie de l’Ouest, Site René Gauducheau, Saint Herblain, France; Department of Medical Oncology, CHU Bretonneau Tours, Tours, France; Department of Gynecology, CHU Bretonneau Tours, Tours, France; Department of Medical Oncology, CHU Bretonneau Tours, Tours, France; Department of Medical Oncology, Institut de Cancérologie de l’Ouest, Site Paul Papin, Angers, France; Data Factory and Analytics, Institut de Cancérologie de l’Ouest, Site René Gauducheau, Saint Herblain, France; Department of Medical Oncology, Institut de Cancérologie de l’Ouest, Site René Gauducheau, Saint Herblain, France; Department of Medical Oncology, CHU Bretonneau Tours, Tours, France

**Keywords:** ovarian epithelial carcinoma, aromatase inhibitors, estrogen receptor antagonists, platinum sensitivity

## Abstract

**Background:**

In women, ovarian cancer is the eighth most frequent cancer in incidence and mortality. It is often diagnosed at advanced stages; relapses are frequent, with a poor prognosis. When platinum resistant, subsequent lines of chemotherapy are of limited effect and often poorly tolerated, leading to quality of life deterioration. Various studies suggest a hormonal role in ovarian carcinogenesis, with a rationale for endocrine therapy in these cancers.

**Patients and Methods:**

This multicenter, retrospective study assessed the use of endocrine treatment for high-grade ovarian epithelial carcinomas treated between 2010 and 2020.

**Results:**

Eighty-one patients with ovarian cancers were included. The median duration of platinum sensitivity was 29 months. We observed a 35% disease control rate with endocrine therapy, and 10% reported symptom improvement. For 19 patients (23.5%), the disease was stabilized for more than 6 months. Median overall survival from diagnosis was 62.6 months. Regarding endocrine therapy predictive factors of response, in a multivariate analysis, 3 factors were statistically significant in favoring progression-free survival: platinum sensitivity (*P* = .021), an R0 surgical resection (*P* = .020), and the indication for hormone therapy being maintenance therapy (*P* = .002)

**Conclusion:**

This study shows real-life data on endocrine therapy in ovarian cancer. As it is a low-cost treatment with many advantages such as its oral administration and its safety, it may be an option to consider. A perspective lies in the search for cofactors to aim as future therapeutic targets to improve the effectiveness of hormone treatment by means of combination therapy.

## Teaser

Some studies suggest a hormonal role in ovarian carcinogenesis, with a rationale for endocrine therapy in these cancers. This multicenter retrospective study analyzed patients with ovarian cancers expressing hormonal receptors to determine the feasibility of endocrine therapy.

**Implications for Practice**

Various studies suggest a hormonal role in ovarian carcinogenesis, with a rationale for endocrine therapy in these cancers. This multicenter retrospective study on 81 patients with ovarian cancers expressing hormonal receptor, shows that endocrine therapy is feasible with some efficacy. As it is a low-cost treatment, well tolerated, it may be an option to consider in ovarian cancer, particularly for patients not eligible for other standard treatments such as chemotherapy or PARP inhibitors.

## Background

Ovarian cancer is the most lethal gynecologic cancer, relative to incidence, in developed countries. In France, in 2018, the incidence was approximately 4600 new cases and 3000 deaths per year.^1,2^ Despite complete remissions obtained after surgery and chemotherapy, recurrences are unfortunately frequent. The 5-year recurrence rate is 75% for high-grade serous ovarian carcinomas with a median delay of 18 months after the end of treatment.^3,4^ Despite therapeutic advances such as maximal cytoreduction surgery and targeted therapies, overall survival (OS) at 5 years remains low, estimated around 40%.^1^

- After initial chemosensitivity to platinum salts, the disease often becomes resistant. This secondary resistance is defined by disease progression within 6 months after the last platinum salt chemotherapy administration. Subsequent lines of platinum-free chemotherapy are often poorly tolerated and inefficient with a median OS of approximately 15 months.^5^ In this context, it is difficult to determine the optimal treatment and when exclusive comfort care would be preferable to a new line of “active” therapy.

There is also considerable evidence to suggest a role for hormones, particularly estrogen, in ovarian cancer. This cancer is more frequent in women who experienced early menarche or late menopause, those who have had few or no children, and those who gave birth at an advanced age.^6^ These events are associated with increased exposure to estrogen; thus, estrogen has been implicated as a risk factor for ovarian cancer.^6^

This role of estrogen was also highlighted by a meta-analysis of 52 epidemiological studies of long-term use of hormone replacement therapy in postmenopausal women. This study identified an increased relative risk (RR) of ovarian cancer of 1.20 (95% CI, 1.15-1.26; *P* < .0001) for long-term users compared with non-users.^7^

More than 80% of high-grade serous or endometrioid carcinomas of the ovary express hormone receptors, in particular, the estrogen receptor (ER) Erα.^8,9^ Experimental data show the activity of estrogen on ovarian cancer cell proliferation in cell culture and in a xenograft model.^9,10^ The activity of aromatase inhibitors on ovarian cancer cells expressing ERα has been demonstrated in a mouse model.^11^

Hormone therapy offers an attractive treatment option for these patients because of the convenience of oral administration and a more favorable safety profile compared with chemotherapy and other targeted therapies.

Even if endocrine therapy is not EMA approved for ovarian cancer, the National Comprehensive Cancer Network guidelines (version 3.2012) classified it as “other potentially effective drugs” for recurrent epithelial ovarian cancer.^12^ The 2019 EMSO-ESGO consensus conference estimated a grade B in evidence level concerning the predictive role of moderately to highly expressed ERs for response to endocrine therapy.^5^

A Swedish cohort study found that only 1 patient out of 5 with epithelial ovarian cancer received endocrine therapy during disease treatment.^13^ It could nevertheless still be an interesting treatment option thanks to its low toxicity risk, even if its efficacy remains debatable.

This multicenter retrospective observational study aimed to address this issue in a real-life setting.

## Methods

### Study design

This noninterventional, multicenter, retrospective study aimed to describe the outcome with endocrine therapy in high-grade ovarian cancer within 2 centers (Tours University hospital, Institut de Cancérologie de l’Ouest Saint-Herblain and Angers). The study was conducted in accordance with the Declaration of Helsinki, and the protocol was approved by the Ethics Committee of Tours. This study was registered in HDH (study number F20210121142805), in accordance with French legislation and CNIL (“Commission nationale de l’informatique et des libertés”). Non-opposition and consent letters were sent to patients and the study was handled according to the data protection officers’ requirements.

In the present study, we selected patients who had high-grade epithelial carcinoma of the ovary, fallopian tubes, or primary peritoneal carcinoma, who had received at least one line of endocrine therapy during follow-up: aromatase inhibitor (AI) or anti-estrogen (tamoxifen, fulvestrant, and megestrol acetate). Patients with low-grade ovarian carcinoma, ovarian sex cord tumor, and those who had received endocrine therapy for breast cancer were excluded. Demographic, clinicopathological characteristics, treatment modalities, and outcomes were recorded from patients’ electronic medical records. Patients had a CT scan every 3 months to evaluate response to endocrine therapy, as per standard procedure in the courant clinical practice of both centers.

Patients were considered to have platinum-resistant disease when they had progressive disease in less than 6 months after their last chemotherapy with platinum salts.

### Objectives and endpoints

The primary endpoint was to evaluate the disease control rate (DCR) after 3 months of hormone therapy. DCR was defined as response or stability according to RECIST1.1 criteria. Secondary endpoints were progression-free survival (PFS), defined as the interval between the date of initiation of endocrine therapy and the first progression or death, and overall survival (OS), defined as the interval between cancer diagnosis and death. We also characterized patients whose disease was stabilized for more than 6 months with endocrine therapy, called “long responders,” to identify predictive factors for endocrine therapy efficacy.

### Statistical analysis

Median PFS and OS were estimated with the Kaplan-Meier method. Univariable analysis was performed to identify the prognostic factors associated with PFS. The variables with a *P* < .2 in univariate analyses were included in the primary model for the multivariate analysis. Multivariable adjusted hazard ratios (HRs) were estimated with Cox proportional hazards models. All statistical tests were 2-sided and *P*-values < .05 were considered statistically significant. Statistical analyses were performed by using SPSS (Statistical Package for the Social Sciences) version 26.

## Results

### Patient’s characteristics

A total of 81 patients were included in the study. The initial characteristics of the patients are presented in Table 1.

**Table 1.** Patient characteristics.

	Overall population *n* = 81
Age at diagnosis (years), median (min-max)	65 (31-91)
FIGO stage, *n* (%)	
IA-IC	2 (2.5)
IIA-IIC	2 (2.5)
IIIA-IIIB	8 (9.9)
IIIC	53 (65.4)
IV	16 (19.7)
Initial localization, *n* (%)	
Ovary	59 (72.8)
Fallopian tube	1 (1.2)
Peritoneum	6 (7.4)
Undetermined	15 (18.5)
Histology, *n* (%)	
Serous high-grade carcinoma	61 (75.3)
Endometrioid carcinoma	2 (2.5)
Mucinous carcinoma	1 (1.2)
Seromucinous carcinoma	11 (13.6)
Undifferenciated carcinoma	6 (7.4)
Hormonal receptors expression, *n* (%)	
ER− PR−	1 (1.2)
ER+	51 (63)
ER− PR+	1 (1.2)
Analysis not performed	28 (35.6)
BRCA status, *n* (%)	
Mutation	2 (2.5)
Wild type	33 (40.7)
Analysis not performed	46 (56.8)
Surgery, *n* (%)	
Frontline	34 (42.0)
Interval	35 (43.2)
Never performed	12 (14.8)
Resection margins, *n* (%)	
R0	39 (56.5)
R1	14 (20.3)
R2	13 (18.8)
Unknown	3 (4.3)
Exposition to platinum salts, median (min-max)	
Number of cycles of chemotherapy before endocrine therapy	12 (0-36)

FIGO stage: Classification established by the International Federation of Gynecology and Obstetrics.

Abbreviations: ER, estrogen receptor; PR, progesterone receptor; BRCA status, BReast Cancer.

Briefly, the median age was 65 (31-91) years, and the disease was diagnosed at an advanced stage (FIGO III-IV) in most patients. Debulking surgery was performed in 69 patients (85%). Expression of hormonal receptors on tumor were positive in 52/53 (98.1%). The *BRCA* mutation status and HRD status were known in less than half of the cases. The median number of previous lines of chemotherapy was 4 (0-12) and 14.8% of patients had received bevacizumab in maintenance therapy. No patient received PARP inhibitor in maintenance therapy. At the initiation of endocrine therapy, 24/78 (30.8%) and 54/78 (69.2%) of patients had platinum-sensitive and platinum-resistant disease, respectively. Endocrine therapy was given as a proper line of treatment in 60/81 (74.1%) or as maintenance therapy after chemotherapy in 21/81 (25.9%) of patients. Aromatase inhibitor and tamoxifen were mainly prescribed to 37/81 (45.7%) and 40/81 (49.4%) of patients, respectively. The 4 remaining patients received fulvestrant or acetate of megestrol. Post-endocrine therapy progression treatments were delivered in 53/81 (65.4%) of patients with a median number of 1 (0-11) including further lines of endocrine therapy in 19/53 (35.8%) patients.

### Tolerance and efficacy of endocrine therapy

With a median follow-up of 59 (3-268) months from ovarian cancer diagnosis, the median duration of endocrine therapy was 3 (0.4-87.7) months. At the 3-month evaluation, 65/81 patients (80.2%) reported good tolerance of endocrine therapy, 4 patients (4.9%) had grade 1/2 arthralgia. Two patients switched to another type of hormone treatment for toxicity reasons.

No patient had a complete response and only one had a partial response. This patient was treated with tamoxifen. However, stable disease was achieved as the best response in 27/81 (33.3%) of patients, including 12 and 15 patients treated with aromatase inhibitors and tamoxifen, respectively. This led to a 35% (28/81) disease control rate. Median PFS was 3 (95% CI, 2.9-3.1) months. Patients who received endocrine therapy as a line of treatment versus as a maintenance therapy had a median PFS of 3 (95% CI, 2.6-3.4) and 4.9 (95% CI, 3.4-6.4) months, respectively.

Regarding the reasons for endocrine therapy initiation, in the overall population, 49 patients (60.5%) had progressive disease, 29 (35.8%) were in a post-chemotherapy maintenance setting, and 3 (3.7%) received endocrine therapy as their first line of systemic therapy because of contraindication to chemotherapy.

### Long responders and multivariate analyses of benefit of endocrine therapy

We identified 19 patients (23.5%) as “long responders,” ie, whose disease remained stable for more than 6 months on the first use of endocrine therapy. Their median age was 63.8 (37-86) years, and 16/19 (84.2%) had serous high-grade carcinomas. Hormonal receptors were expressed in tumors for 14/15 (93.3%) patients. None of the patients with germline testing carried a BRCA mutation (0/9). These patients had received a median of 3 previous lines of chemotherapy and were more likely to have platinum-sensitive disease at initiation (58% vs 30% for no-long responders). Their median duration of platinum sensitivity was also longer (40.5 months) than for the global population (29 months). Interestingly, 12/19 (63.2%) of these patients received endocrine therapy in a maintenance setting. Median OS from diagnosis was 62.6 (95% CI, 52.5-72.7) months for the overall population and 68.5 (95% CI, 58.4-78.6) months for the long responders.

After univariate and multivariate analyses using the Cox regression model, 3 factors were statistically significantly correlated to progression-free survival when treated with endocrine therapy: the platinum sensitivity (HR = 0.52; 95% CI, 0.29-0.90; *P* = .021), an R0 surgical resection (HR = 0.56; 95% CI, 0.34-0.91; *P* = .020), and the setting of initiation endocrine therapy, ie maintenance or prior progression (HR = 0.36; 95% CI, 0.19-0.68; *P* = .002), as reported in Table 2.

**Table 2.** Cox univariate and multivariate analyses of progression-free survival.

	Univariate analysis		Multivariate analysis	
	HR [95% CI]	*P*	HR [95% CI]	*P*
Platinum sensitivity (yes vs no)	0.47 [0.28-0.80]	.005	0.52 [0.29-0.90]	.021
Residual disease (R0 vs R+)	0.61 [0.38-0.96]	.032	0.56 [0.34-0.91]	.020
Number of lines of treatment before endocrine therapy (≤4 vs >4)	0.82 [0.52-1.39]	.397	—	—
Type of endocrine therapy (AI vs AE)	1.07 [0.69-1.66]	.772	—	—
Setting of initiation of endocrine therapy (maintenance therapy vs progressive disease)	0.35 [0.19-0.65]	.001	0.36 [0.19-0.68]	.002

Abbreviations: AI, aromatase inhibitor; AE, anti-estrogen.

## Discussion

In this study, 95% of the patients were diagnosed with advanced disease. The median age at diagnosis was 65 years. Most patients had received multiple lines of therapy prior to endocrine therapy. The majority (66.7%) had platinum-resistant disease at the initiation of endocrine therapy. The disease control rate at 3 months was 35%, consisting of stabilized disease with the exception of one partial response obtained.

George et al^14^ found similar results in a larger cohort of 97 patients. The 2 populations had common characteristics: median age at diagnosis was 63 years, they too had received multiple prior therapies with a median of 3 lines, and 40% had platinum-sensitive disease. In this study, it was also found that endocrine therapy could stabilize the disease, with 45% of patients having stable disease for more than 3 months on endocrine therapy, the median duration of stabilization was 9.6 months with letrozole and 7.2 months with tamoxifen.

In the Bagge et al^13^ study, the median age of the patients was also 63 years, and the diagnosis was often made at an advanced stage (77% FIGO stage IIIc-IV). However, they were much less pretreated before hormone therapy, as 50% of patients had received only one line of treatment at the initiation of hormone therapy and less than 20% had received 3 or more lines of treatment. In addition, 57% of patients had responded to the last line of treatment received and therefore received hormone therapy for maintenance purposes. This may explain a better response to hormone therapy with 5% partial response and 50% stable disease after 3 months of treatment.

Several phase 2 studies, as well as some retrospective studies, have also shown a possible benefit of endocrine therapy (Table 3). A meta-analysis of 53 trials and 2490 patients treated for all grades of epithelial ovarian carcinoma found a clinical benefit for 41% of patients: 55% in the platinum-sensitive tumors versus 40% in the resistant ones.^16^ In several phase 2 trials, with small enrollment, but including only patients treated for high-grade epithelial ovarian carcinoma, clinical benefit was highly variable, measured between 28% and 83%, with a median progression-free survival (PFS) ranging from 2 to 12.5 months (Table 3).

**Table 3.** Literature review.

Reference	Study type	*N*	Indication	Platinum resistant	HR status	Endocrine Therapy	PFS (months)	CR (%)	PR (%)	SD (%)	DCR (%)
Paleari^15^	Meta-analysis	2490	Progression	—	—	—	—	—	—	—	41
Hatch^16^	Phase 2	105	Progression or Maintenance	—	HR+/−	Tamoxifen	7.5	10	8	38	52
Markman^17^	Phase 2	102	Progression or Maintenance	79	—	Tamoxifen	—	10	7	—	—
Tropé^18^	Phase 2	66	Progression	100	—	Tamoxifen	4	3	3	77	83
Bowman^19^	Phase 2	60	Biological progression	—	HR+/−	Letrozole	—	0	9	26	35
Wagner^20^	Phase 2	56	Progression	100	—	Tamoxifen	2	0	0	33	33
Kok^21^	Phase 2	54	Biological progression	—	HR+	Anastrozole	2.7	0	4	30	35
Del Carmen 2003^22^	Phase 2	53	Progression	—	HR+/−	Anastrozole	2.8	—	2	42	—
Heinzelmann^23^	Phase 2	50	Maintenance	—	HR+	Letrozole	NA	—	—	60	—
Bonaventura^24^	Phase 2	49	Progression	100	HR+	Anastrozole	2.7	0	0	28	28
Smyth^25^	Phase 2	42	Progression	43	HR+	Letrozole	26% > 6 5% ≥ 24	0	17	26	51
Weiner^26^	Phase 2	37	Progression	—	HR+/−	Tamoxifen	—	3	6	19	29
Ramirez^27^	Phase 2	33	Progression	100	HR+	Letrozole	2	0	3	21	26
Ahlgren^28^	Phase 2	29	Progression or Maintenance	39	—	Tamoxifen	12.5	—	—	—	17
Papadimitriou^29^	Phase 2	27	Progression	33	HR+/−	Letrozole	—	5	10	19	33
Argenta^30^	Phase 2	26	Progression	—	HR+	Fulvestrant	2	0	0	50	50
Hasan^31^	Phase 2	26	Progression	65	—	Tamoxifen and goserelin	4	4	8	38	50
Verma^32^	Phase 2	22	Progression	—	HR+/−	Exemestane	2	0	0	36	36
Stanley^33^	Retrospective	269	Progression	13	HR+/−	Letrozole or tamoxifen	4	3	5	32	40
Bagge^13^	Retrospective	248	Progression	—	HR+/-	Tamoxifen or AI	4	0	5	49	54
Stasenko^34^	Retrospective	99	Progression	100	HR+/−	Tamoxifen or AI	4	—	—	—	—
George^14^	Retrospective	97	Progression	60	HR+/−	Letrozole or tamoxifen	10	0	14	45	59
Karagol^35^	Retrospective	29	Progression	100	—	Tamoxifen	4	3	7	21	31

Abbreviations: PFS, progression-free survival; NA, not achieved; CR, complete response; PR, partial response; SD, stable disease; DCR, disease control rate; HR, hormone receptor; AI, aromatase inhibitor; “—”: missing data.

Bowman et al^19^ demonstrated that letrozole was active in epithelial ovarian carcinoma expressing a high level of ER, which led to stabilization and biological response on CA125 (Cancer Antigen 125), suggesting endocrine therapy sensitivity. Endocrine therapy may be effective in biological recurrence with elevated CA125 with no indication for chemotherapy initiation; a phase 2 study using anastrozole in this situation found a clinical benefit in 34.6% and 6.5 months median duration of this clinical benefit.^22^

The phase 3 trial, Ovaresist, randomized 283 patients with platinum-resistant ovarian cancer to chemotherapy (with paclitaxel or pegylated doxorubicin) or endocrine therapy with tamoxifen.^36^ PFS was 12.7 weeks in the chemotherapy arm (95% CI, 9.0-16.3) versus 8.3 weeks in the tamoxifen arm (95% CI, 8.0-10.4). Yet, even if the difference was statistically significant (*P* = .003), patients on chemotherapy experienced more toxicity, reported poorer quality of life, particularly with no better control on their digestive symptoms, thus questioning the real benefit of chemotherapy.

Recurrence of ovarian cancer remains an incurable disease and therefore stabilization of the disease should be considered an important therapeutic outcome, especially in those heavily pretreated, who have exhausted standard lines of treatment or who wish to delay a further line of chemotherapy to preserve their quality of life. In our study, it is important to note that 66.7% of patients received further lines of treatment after endocrine therapy, in many cases due to stabilization of symptoms and improvement in general condition. We found this interesting as the study was conducted in a real-life setting, with recent data, as the patients were included in the last 10 years.

We observed that the patients that received endocrine therapy longer had distinctive characteristics as most of them had had long platinum sensitivity and/or were still platinum sensitive, and had received a median of 3 previous lines of chemotherapy. This suggests that those patients probably had a particular form of ovarian cancer, even if 84% had serous high-grade carcinomas, the disease seems to be biologically less aggressive than usually expected.

There were several limitations to our study due to its retrospective nature: imaging reassessment during endocrine therapy was performed irregularly compared to reassessments during chemotherapy performed every 3 cycles. The reason to stop endocrine therapy was not always reported in the medical record. The extent of symptom improvement was difficult to assess as it was filled only in a minority of cases. Tolerance was also not always mentioned.

Our study population was a heterogeneous group of patients, as endocrine therapy was used for different indications. We also need to emphasize that it was mainly prescribed in a maintenance setting for most of the patients who received it during 6 months or more.

As we observed that a subgroup of patients was stabilized more than 6 months with endocrine therapy, we believe that predictive factors for response or cofactors for future therapeutic targets should be identified.

The tumor status of estrogen and progesterone receptors, which have already been shown to play a prognostic role, has previously been evaluated as predictive markers of response to endocrine therapy, including ERα expression or an ER histoscore > 200.^8,9,19,33^ However, although a large proportion of epithelial ovarian cancers express the ERα receptor, this factor alone does not seem to be sufficient to predict response to hormone therapy.

In the study by George et al,^14^ there was no statistically significant difference in objective control rate (ORR) according to tumor hormone receptor status or platinum sensitivity at initiation of endocrine therapy. Concerning the hormone receptor status, it may be explained by the fact that it was only available for 53% of their population. For platinum sensitivity, there was no statistically significant association although the response rate was slightly higher for sensitive diseases (17.9%) than for resistant (12.1%). Our study found a difference in PFS with endocrine therapy according to platinum sensitivity (HR = 0.52; 95% CI, 0.29-0.90; *P* = .021) and other studies support this. Ramirez et al^27^ evaluated letrozole in patients with platinum-resistant ovarian cancer, reporting an ORR of 3%. On the other hand, Papadimitriou et al^29^ evaluated letrozole in a cohort of ovarian cancer patients, 67% were platinum-sensitive, with an ORR of 15%. The platinum-sensitive disease is considered less aggressive than platinum-resistant disease. The impact of platinum sensitivity on the clinical benefit of endocrine therapy needs to be further studied.

The reason for initiating endocrine therapy is also correlated with endocrine therapy PFS. This is easily explained since the disease is more aggressive when it is progressive and so more difficult to control than in a maintenance setting.

Similarly, surgical margins are a usual prognostic factor, and we found here that it was still verified when treated with endocrine therapy.

Several studies have looked for predictive factors of response to endocrine therapy. Bowman’s trial found histological factors that appeared to be associated with a better response to endocrine therapy such as high levels of ER, low levels of HER2 (human epidermal growth factor receptor-2), and high levels of epidermal growth factor receptor.^20^ Another trial found a better response to letrozole if there were low HER2, IGFBP5, and vimentin expressions and high TTF1 expression.^32^ Similarly, Walker’s study found differences in the expression levels of TFF1, TFF3, BIGH3, TRAP1, VIM, TOP2A, PLAU, and UBE2C in tumors that correlated with their response to endocrine therapy.^37^ All these results could help to better identify patients who could benefit from endocrine therapy.

Molecular and clinicopathological data from The Cancer Genome Atlas Program suggest a correlation between ERα and DOT1L (distuptor of telomeric silencing 1-like) expression and prognosis in ovarian cancers.^38^ In the study by Salvati et al,^39^ the key role of DOT1L was demonstrated. It has a role in functional cooperation with ERα and an epigenetic role in the transmission of estrogen signaling to modulate the transcription of genes involved in cell proliferation. Furthermore, these results provide evidence suggesting that combined inhibition of these 2 factors (ERα/DOT1L) may be effective in blocking estrogen signaling in chemoresistant ovarian tumors.

In a recent online survey, patients with ovarian cancer were asked about their goals of care and priorities.^40^ The goals of these treatments changed with time, probably due to disease evolution. This survey of 328 patients reported that they were willing to risk serious treatment-related side effects when the goal of treatment was curative, but much less when the goal of treatment was only to stabilize the disease. It also showed that 45% of patients set their main goal of treatment to improve survival, 41% to improve quality of life, and 12% to stabilize the disease. Thus, it seems important that oncologists carefully explain the goals of treatment to the patient and consider the patient’s priorities to determine the best treatment option with her.

In an English retrospective study published in 2011, in 274 patients followed for platinum-resistant ovarian cancer, the median OS since initiation of first-line treatment for platinum-resistant disease was 14 months.^5^ For 28% of patients, a new line of chemotherapy had been initiated within 3 months prior to death. This study concluded that if disease control was not achieved after 2 lines of treatment for platinum-resistant disease, subsequent lines were not more effective, with only 5.1% of clinical benefit in the third line.

In this situation of advanced and refractory disease, discontinuation of specific treatments may also be considered, given the lack of clear scientific evidence of the efficacy of any treatment at this stage. Overall management, apart from the specific treatment, must be anticipated, to avoid this announcement being synonymous with a cessation of care for the patient.

Helft’s connivance strategy should be used as early as possible to communicate with the patient, as it allows to give information in terms of possible future events: anticipating the occurrence of negative events (the shorter and shorter chemotherapy responses, and the fact that chemotherapy may one day no longer work) and maintaining hope with possible events under treatment (the improvement in general condition, the decrease in disabling symptoms).^41^ In this way, honest uncertainty about the course of disease and its prognosis will be useful to the patient’s hope in leaving an open ending.

## Conclusion

Hormone therapy in our study was used for one-third of the cases in a maintenance setting and for the other two-thirds for progressive disease.

The main limitation of this retrospective study is the heterogeneity of the population (platinum sensitive or not, different indications for initiation of endocrine therapy). Hormone therapy has such advantages: ease of administration, low cost, and low risk of toxicity; given the benefit induced in a subgroup of patients, it would be interesting to identify predictive biomarkers of response to hormone therapy or to find cofactors that could be future therapeutic targets to use them more widely, and wisely, in clinical practice.

## Data Availability

The datasets used and/or analyzed during the current study are available from the corresponding author on reasonable request.

## Abbreviations

AI: aromatase inhibitors
BRCA: BReast Cancer gene
CA125: cancer antigen 125
CNIL: “Commission Nationale de l’Informatique et des Libertés”
DCR: disease control rate
DOT1L: distuptor of telomeric silencing 1-like
EGFR: epidermal growth factor receptor
ERα: estrogen receptor alpha
FIGO: International Federation of Gynecology and Obstetrics
HER2: human epidermal growth factor receptor-2
HR: hazard ratio
NK: not known
ORR: objective response rate
OS: overall survival
PFS: progression-free survival
PM: disease progression
PR: partial response
RR: relative risk
SPSS: Statistical Package for the Social Sciences
TCGA: The Cancer Genome Atlas Program
